# Dataset of seismic ambient vibrations from the quaternary Norcia basin (central Italy)

**DOI:** 10.1016/j.dib.2020.105709

**Published:** 2020-05-18

**Authors:** Maurizio Ercoli, Giuseppe Di Giulio, Maurizio Vassallo, Massimiliano Porreca

**Affiliations:** a Dipartimento di Fisica e Geologia, Università degli Studi di Perugia, Via Alessandro Pascoli, 06123 Perugia, Italy; b Istituto Nazionale di Geofisica e Vulcanologia, sezione di Roma1, sede di L'Aquila, viale Francesco Crispi 43, 67100 L'Aquila, Italy

**Keywords:** Seismic ambient vibrations, Active seismic, Seismic array and ambient noise analysis, GIS data, Norcia basin, Site effects

## Abstract

Central Italy was affected by a long seismic sequence in 2016 and 2017, characterized by five main-shocks with Mw>5.0. The Mw 6.5 mainshock occurred on 30 October 2016 close to the town of Norcia, located in the intra-Apennine Norcia basin. Different degrees of damages were observed during this seismic crisis, caused by a variable seismic shaking. This was also due to important 1D and 2D variation of Quaternary fluvio-lacustrine sediments infilling the basin. Following such considerations, a new geophysical dataset of seismic vibration measurements was acquired in the study area during the period April 2017–November 2019. We collected mainly single-seismic station noise data, to infer the distribution of resonance frequency (f_0_) of the basin. A total of 60 sites were measured to cover the entire extension in the basin. We deployed seismometers along three transects of a total length of 21 km, mostly along the main structural directions of the basin (i.e. NNW-SSE and NE-SW). Two 2D arrays of seismic stations with a elicoidal-shaped geometry, and a set of MASW active data were also acquired in the northern sector of the basin, in order to better constrain the seismic velocity of the sedimentary infilling. These new records have been integrated with available geological information in order to reconstruct the deep structure of the basin, as discussed in the research paper by [2]. The entire dataset used in [2] is here provided, together with 7 additional records recovered for the basin (i.e. N54-N60) and ancillary open-source geospatial data. The dataset can be used for different purposes: specific research on the Norcia basin, comparative studies on similar areas around the world, development of new data modeling and testing of new analysis software, and as a training dataset for machine learning applications.

Specifications table

**Table:** 

Subject	Earth and Planetary Science; Geophysics; Geotechnical engineering and Engineering Geology
Specific subject area	Geophysics, seismology
Type of data	Table, figures, text file, digital time-series, geospatial data
How data were acquired	Seismic campaign using different mobile seismographs equipment. Single station equipment, as combination of high-dynamic digitizers and three-component seismometers (Reftek130 digitizer with 5 s Lennartz triaxial velocimeter, two SARA Geobox – 4.5 and 0.5 Hz terns of geophones), and a multi-channel seismic equipment (SARA Do.Re.Mi 12 channels seismograph with 4.5 Hz vertical geophones).
Data format	Raw data Formats:
	(1) SAC (2) SEG-Y (3) Geopackage (4) Keyhole Markup Language
Parameters for data collection	Passive measurements were conducted with single stations and with a sampling rate at least 200 Hz. The sampling rate was 1000 Hz for active data.
Description of data collection	The dataset was acquired during different campaigns from 2017 to 2019. For each passive measurement, the recording time varied from 30 min to 2 h. Passive arrays and active seismic data were collected in the Northern side of the Norcia basin, at the Fontevena and Marcite sites.
Data source location	Norcia basin (Umbria, central Italy) Lat. 42° 47′ 36″ N, Long. 13° 5′ 38″ E
Data accessibility	The entire dataset is published in Mendeley repository. Data identification number: DOI: 10.17632/78pwtzstz6.1 Direct URL to data: https://data.mendeley.com/datasets/78pwtzstz6/1
Related research article	Di Giulio, G., Ercoli, M., Vassallo, M., Porreca, M. (2020). Investigation of the Norcia basin (Central Italy) through ambient vibration measurements and geological surveys. *Engineering Geology, 267*, 105501. https://www.sciencedirect.com/science/article/abs/pii/S0013795219312827

## Value of the data

• Ambient seismic vibrations (noise hereinafter) can be used to determine the properties of the noise wavefield, and compute the resonance frequency (*f*_0_) using *H*/*V* spectral ratios.
• The dataset can help in the reconstruction of the complex stratigraphic architecture and buried substrate of the Norcia basin.
• Data can be cross-checked with numerical data, and used to model active and passive data in a basin environment.
• Researchers, professional geologists and private companies interested in the study of the basin (e.g. seismic response) and post-earthquake recovery of the Norcia area can benefit from these data.
• Future seismic data acquisition can integrate our dataset, to refine the knowledge of the buried geology of the Norcia basin, as well as to improve the understanding of similar basin environments.

## 1 Data

**Fig. 1:**
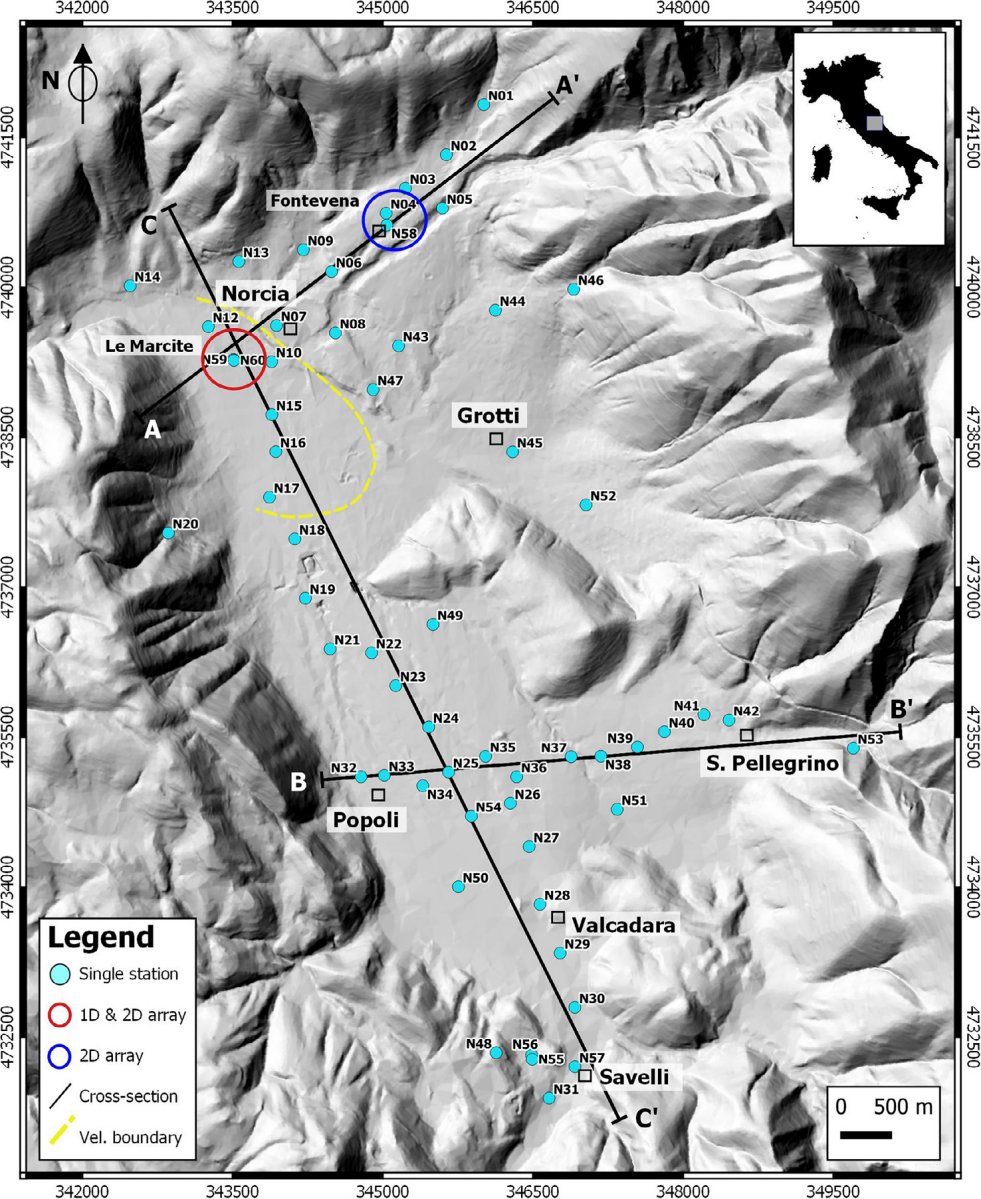
Location map of the study site (Norcia basin). The blue dots display the location of the single-station noise measurements across three main cross-sections (black lines) overlying a high-resolution Digital Elevation Model as basemap [11]. The red and blue circles provide the position of the arrays in the North sector, whilst the yellow dashed is the velocity model boundary used in [2].

The dataset reported in this work mainly consists of ambient vibration measurements carried out in a total of 60 sites covering the intra-mountain basin of Norcia basin in central Italy (Fig. 1). The data were collected during the period 2017–2019, following the 2016–2017 seismic sequence that struck the area. The region is located in the Apennine chain, characterized by a Quaternary extensional tectonic regime, reactivating high-angle normal faults capable of generating earthquakes up to *Mw* = 6.5 [1]. The subsurface architecture of the study area is complex and poorly known due to lack of geophysical data and absence of deep well stratigraphy. This consideration motivated the acquisition of new geophysical data presented here, encompassing seismic records used in [2] and seven additional seismic registrations (Table 1). The seismic recordings are densely distributed across the whole Norcia basin and were collected using pairs of commercial seismic digitizers and velocimeters in similar atmospheric conditions (sunny days with no significant wind). The dataset includes passive single station measurements, 2D arrays, active seismic profiles and georeferenced information which have been accurately organized (see section data assembly) and stored in the Mendeley repository [3].

**Table 1:** The table shows the information of the single-stations (*N*) and the arrays datasets. The table is provided as OpenDocument “.ods” and comma-separated values “.csv” formats in the supplementary material.

Label	Latitude	Longitude	Data filename
*1D single station measurements*			
N01	42.81352	13.11644	171090945_R
N02	42.80893	13.11200	171091007_R
N03	42.80584	13.10710	171091031_R
N04	42.80355	13.10481	171091100_R
N05	42.80411	13.11167	171091121_R
N06	42.79817	13.09831	171091154_R; 171091155_S
N07	42.79321	13.09174	171091423_R
N08	42.79267	13.09895	171091448_R
N09	42.80008	13.09480	171091509_R; 171091514_S
N10	42.78994	13.09124	171100752_R
N11	42.79005	13.08661	171100813_R
N12	42.79297	13.08342	171100830_R
N13	42.79889	13.08696	171100902_R; 171100903_S
N14	42.79648	13.07376	171100927_R
N15	42.78517	13.09141	171100956_R
N16	42.78185	13.09200	171101041_R
N17	42.77773	13.09135	171101059_R
N18	42.77404	13.09456	171101115_R
N19	42.76871	13.09601	171101313_R; 171101319_S
N20	42.77432	13.07911	171101352_R
N21	42.76420	13.09917	181070634_R
N22	42.76393	13.10424	181070657_R
N23	42.76102	13.10728	181070724_R
N24	42.75737	13.11141	181070750_R
N25	42.75335	13.11396	181070813_R
N26	42.75067	13.12158	181071039_R
N27	42.74680	13.12399	181070905_R
N28	42.74163	13.12547	181070930_R
N29	42.73728	13.12806	181070940_R
N30	42.73242	13.13000	181070958_R; 191291712_S
N31	42.72419	13.12711	181071022_R
N32	42.75276	13.10329	181080736_R
N33	42.75293	13.10613	181080750_R
N34	42.75207	13.11085	181080805_R
N35	42.75485	13.11843	181080841_R
N36	42.75307	13.12228	181080736_R
N37	42.75499	13.12887	181080852_R
N38	42.75509	13.13249	181080954_R
N39	42.75598	13.13693	181081025_R
N40	42.75742	13.14018	181080949_R
N41	42.75903	13.14496	181081004_R
N42	42.75859	13.14803	181081027_R
N43	42.79163	13.10669	181061324_R
N44	42.79502	13.11841	181061346_R
N45	42.78231	13.12090	181061304_R
N46	42.79706	13.12791	181061413_R
N47	42.78763	13.10370	181061737_R
N48	42.72818	13.12052	181071307_S
N49	42.76662	13.11166	181071437_R
N50	42.74305	13.11547	181071343_R
N51	42.75033	13.13463	181071405_R; 191291516_S
N52	42.77768	13.13000	181071529_R
N53	42.75631	13.16327	181081317_S
N54	42.74944	13.11686	193331215_S; 193331216_S
N55	42.72766	13.12494	171021214_S
N56	42.72804	13.12486	171021307_S
N57	42.72708	13.13017	171021405_S
N58	42.80248	13.10493	171011218_S
N59	42.79006	13.08656	171011507_S
N60	42.78999	13.08664	191291425_S
*2D arrays*			
FB01	42.80367	13.10574	181100733_R
FB02	42.80385	13.10574	181100744_R
FB03	42.80396	13.10604	181100757_R
FB04	42.80383	13.10644	181100814_R
FB05	42.80346	13.10662	181100824_R
FB06	42.80294	13.10645	181100838_R
FB07	42.80265	13.10529	181100847_R
FB08	42.80266	13.10471	181100857_R
FB09	42.80287	13.10429	181100858_R
FB10	42.80417	13.10365	181100843_R
FB11	42.80514	13.10433	181100831_R
MB01	42.79024	13.08664	181090703_R
MB02	42.79042	13.08664	181090717_R
MB03	42.79058	13.08699	181090734_R
MB04	42.79029	13.08737	181090735_R
MB05	42.79001	13.08758	181090750_R
MB06	42.78950	13.08735	181090759_R
MB07	42.78915	13.08663	181090804_R
MB08	42.78922	13.08562	181090819_R
MB09	42.78980	13.08477	181090818_R
MB10	42.79074	13.08455	181090830_R
MB11	42.79116	13.08574	181090840_R
FS01	42.80367	13.10574	181100733_R
FS02	42.80376	13.10574	181101256_R
FS03	42.80381	13.10588	181101302_R
FS04	42.80375	13.10609	181101334_R
FS05	42.80355	13.10620	181101319_R
FS06	42.80330	13.10610	181101329_R
FS07	42.80313	13.10574	181101339_R
FS08	42.80316	13.10523	181101343_R
FS09	42.80345	13.10481	181101347_R
FS10	42.80392	13.10469	181101352_R
FS11	42.80435	13.10501	181101356_R
MS01	42.79024	13.08664	181090703_R
MS02	42.79033	13.08664	181091154_R
MS03	42.79038	13.08678	181091149_R
MS04	42.79032	13.08700	181091203_R
MS05	42.79012	13.08711	181091215_R
MS06	42.78987	13.08700	181091214_R
MS07	42.78970	13.08664	181091230_R
MS08	42.78973	13.08613	181091228_R
MS09	42.79002	13.08570	181091243_R
MS10	42.79055	13.08560	181091237_R
MS11	42.79097	13.08593	181091249_R

## 2 Experimental design, equipment and data assembly

### 2.1 Experimental design

**Fig. 2:**
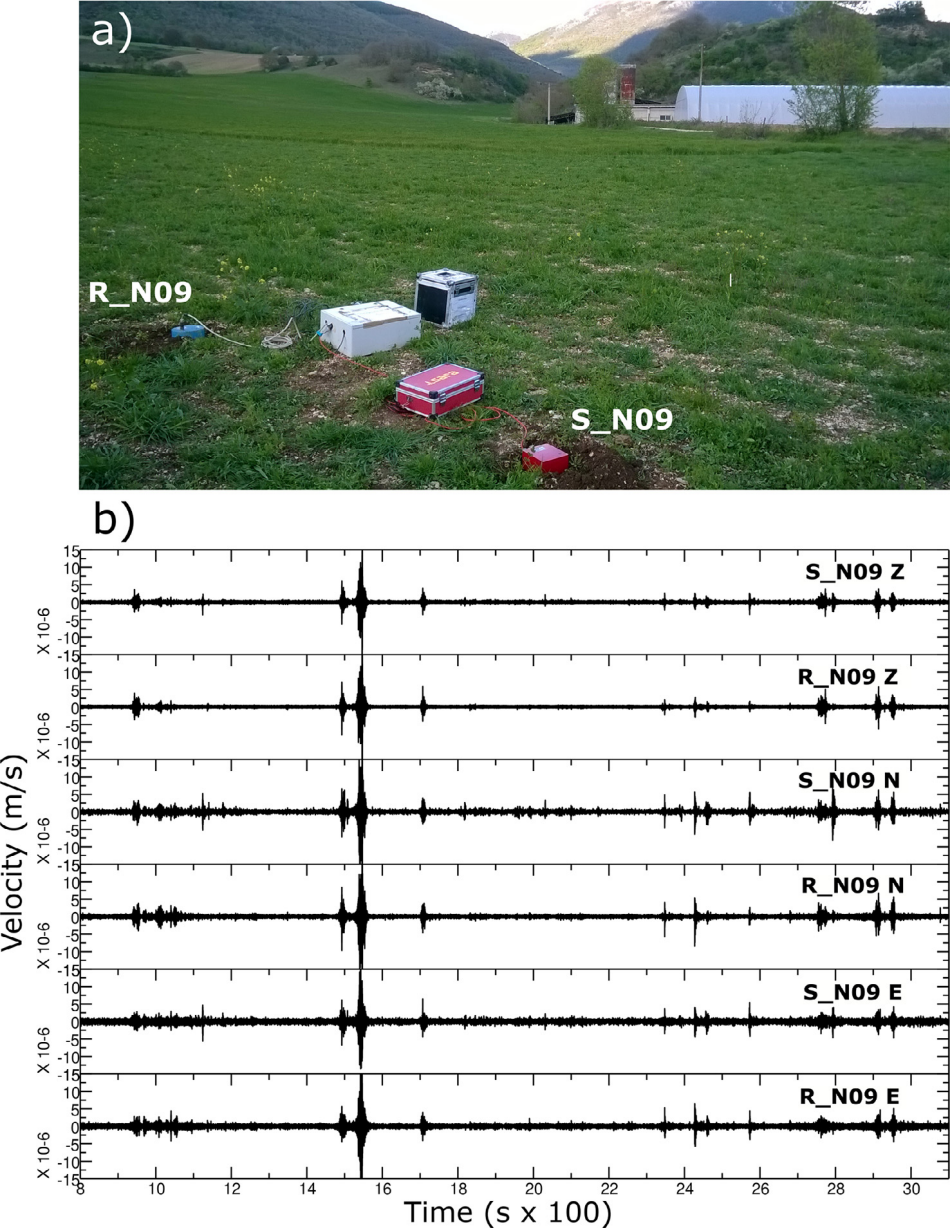
An example of a single-station station measurement (N09 site) at the Norcia basin; (a) contemporary acquisitions carried-out with two different seismic equipment; (b) comparison of the recorded time-series: the Sara Geobox (_S) XYZ traces are displayed close to the corresponding three Reftek (_R) records.

A total of 53 sites of seismic vibration measurements were carried out during the fieldwork [2], whereas 7 additional sites have been added in the present paper. The strategy of the seismic measurements was designed in order to cover the whole Norcia basin. In particular, three main transects were planned along the structural orientation of the tectonic-controlled basin (Fig. 1). The longer transect (9 km long) was planned along the NNW-SSE direction, which is the elongation direction of the basin, parallel to the trend of the main fault (Norcia-Nottoria-Preci fault). Other two minor transets (ca. 4 km long) were designed along the NE-SW and W-E direction in the northern and southern sector of the basin, respectively (Fig. 1). Additional acquisitions were carried out on the uncovered sectors and in correspondence of the borders of the basin. As example Fig. 2 shows the N09 measurement at the northern sector of the basin close to the carbonate substrate.

**Fig. 3:**
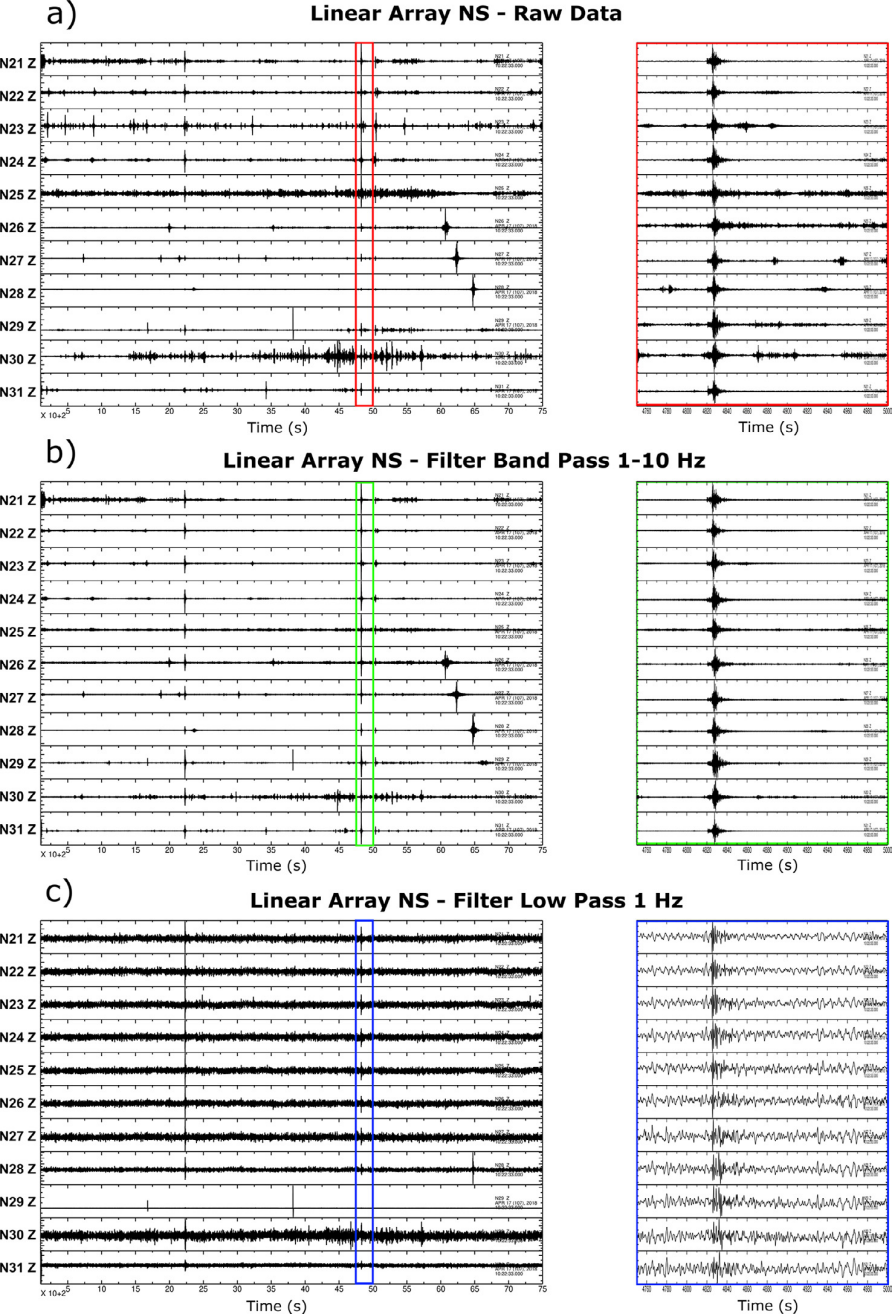
Signals recorded by the vertical components of stations of NW oriented linear array in the southern part of Norcia basin. (a) About 2 h of raw signal acquired by the different stations. In the red box there are seismic events recorded during the acquisition time window. In order to better emphasize the similarities and differences between the signals recorded at the different stations in (b) and (c) the seismic signals are filtered using a band-pass filter from 1 Hz to 10 Hz and a low pass filter at 1 Hz, respectively.

The eleven stations between N21–N31 and the ten stations between N32–N41, installed respectively along a N–S and W–E oriented transects in the southern part of basin, acquired the seismic noise simultaneously for about 2 h. For this reason, such stations can be treated as two linear arrays in passive acquisition whose data are potentially useful for obtaining information on the velocity model in the southern part of Norcia basin (Fig. 3). A possible approach to derive the 1D model from these data is that of ambient noise cross-correlation analysis, as done in the works [2,4].

**Fig. 4:**
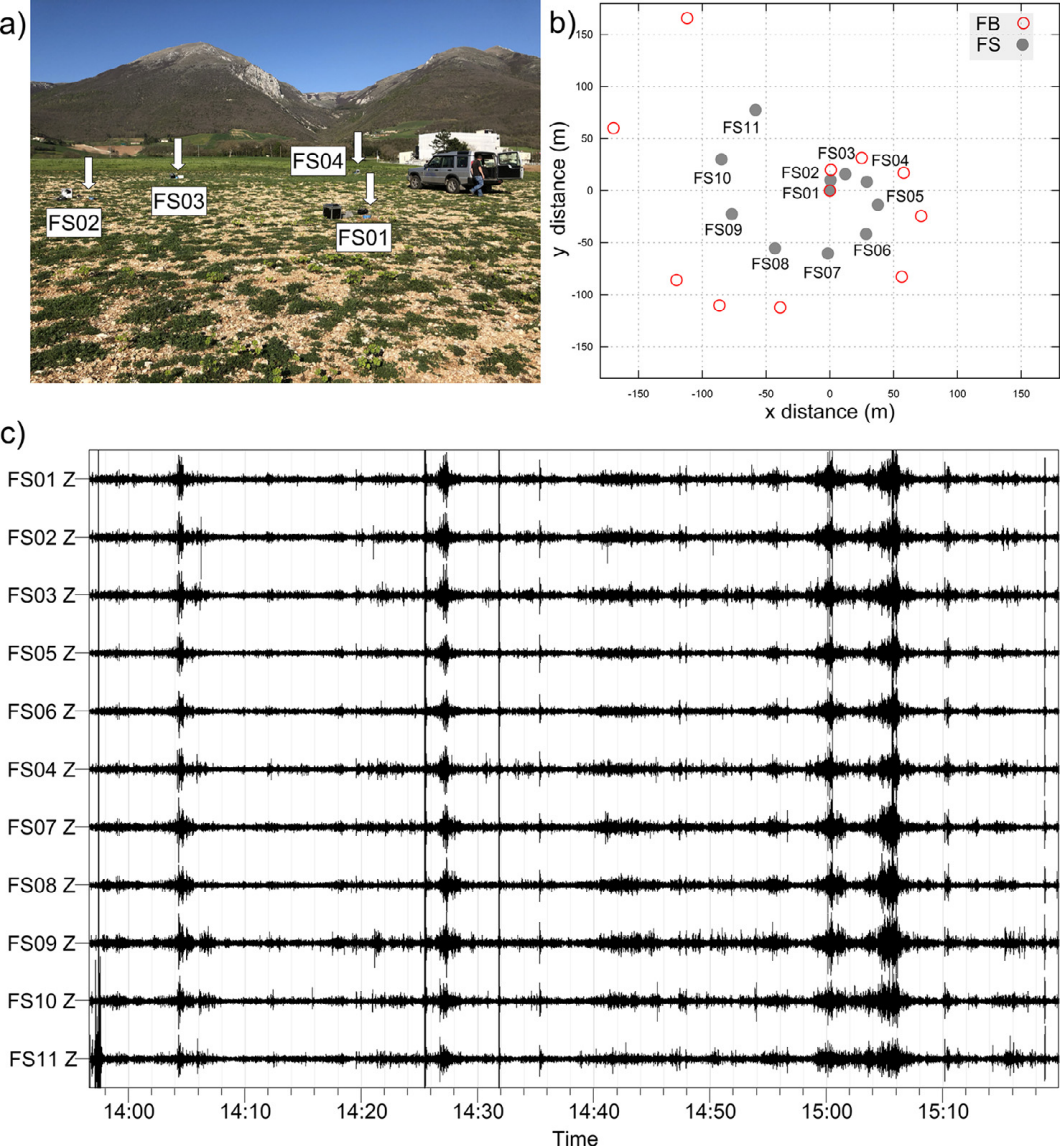
2D array of seismic stations collected at the Fontevena site; (a) picture displaying the position of four stations during the deployment of FS array; (b) geometry of the two arrays FS and FB; (c) portion of the recorded time-series for all the eleven seismic stations of FS array (Z component).

Four 2D helical passive arrays have been performed at two sites in the northern part of Nb (Fig. 1): in the palustrine area named “Marcite” and in the old alluvial fan sector known as “Fontevena”. For each array we deployed 11 seismological stations arranged on a helical geometry (Fig. 4). The seismic stations were synchronized using the GPS antenna receivers. In both the sites, we have designed one minor array (about 150 m of maximum aperture) and a larger one (250 and 300 m of maximum aperture for Marcite and Fontevena, respectively). The 2D arrays were named FB and FS (for Fontevena Big and Fontevena Small), and MB and MS (Marcite Big and Marcite Small). The joint surface-wave inversion shown in Ref. [2] in the Marcite area was done using the mean HV curve computed with the recordings of the MS array. For the Fontevena site, this process was done using N04, very close to FS09 station of the FS array, as a representative HV curve. This decision was taken due to a suspect bias at low-frequency (< 1 Hz) occurred during recording of the FB and FS arrays.

**Fig. 5:**
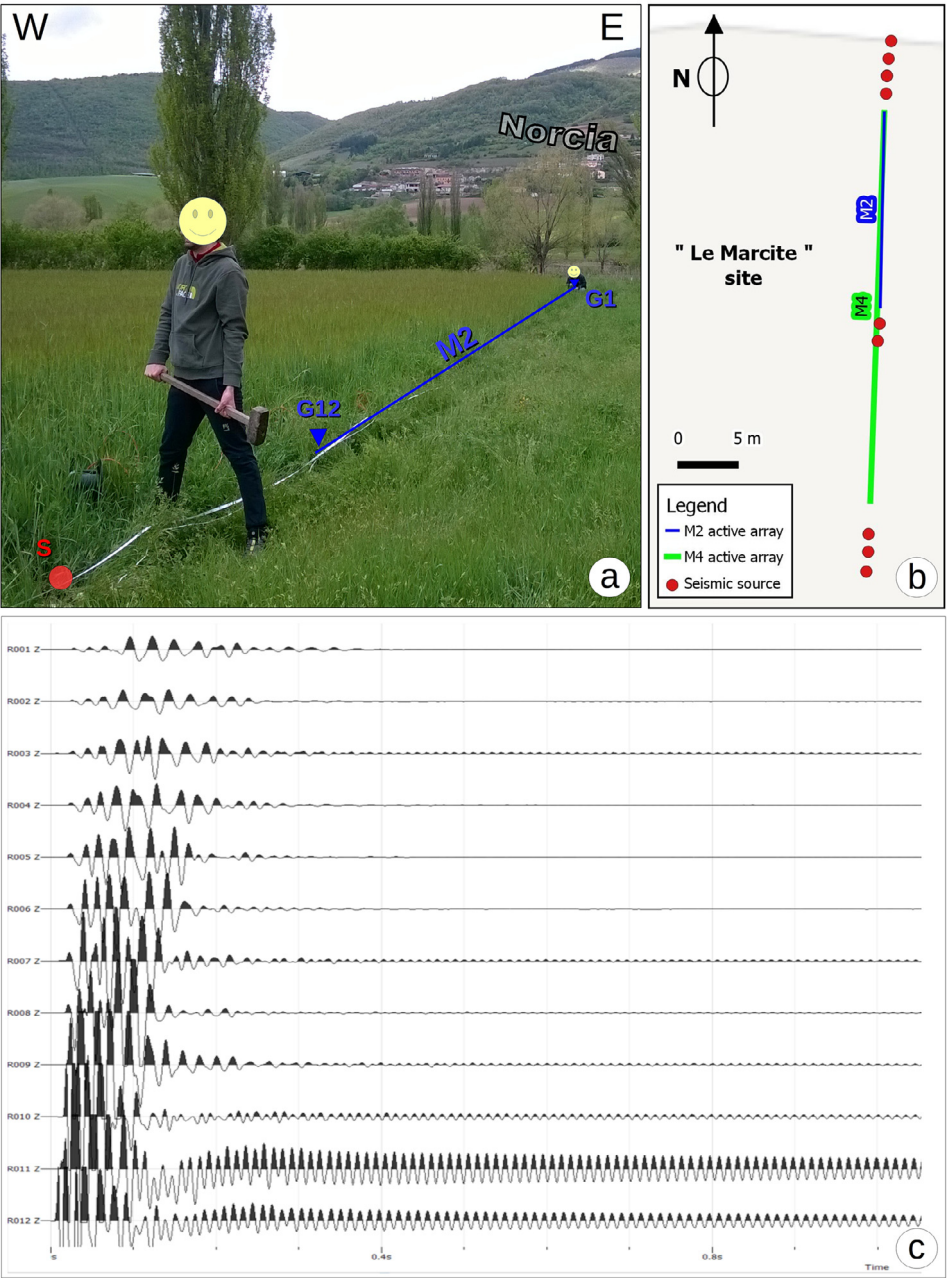
Linear array acquisition at the Marcite site; (a) picture displaying the energization located at the south side of M2; (b) geometry of both M2 and M4; (c) seismogram of the 12 traces recorded (M2_24 file).

Active multichannel seismic records were also registered at the Marcite site (Fig. 5), close to the arrays and to the single-station measurements N11, N59 and N60. The survey was carried out using a “Do.Re.Mi” seismograph (Sara Electronic Instruments s.r.l.) equipped with 12 channels linked to vertical 4.5 Hz geophones (Sara Electronic Instruments s.r.l.). The data have been recorded using two different linear configurations of geophones named “M2” and “M4” (22 m and 44 m long respectively). In the first case M2, we used a geophone spacing of 2 m, and four energizations generated though vertical impacts on a metallic plate, using a 5 Kg sledge-hammer. For M2, two shots were done on the North side close to the geophone 1 (G1), and other two on the South side close to the geophone 12 (G12), with minimum offsets of 2 and 4 m respectively (Fig. 5). In M4 we increased the geophone spacing up to 4 m, using six source points with an offset of 4, 6 and 8 m on either North and South sides (close to G1 and G12 geophones). Table 2 summarizes all the operative parameters related to the offset, geophone spacing and filenames. Each common shot gather encompasses 12 seismic traces, and was collected using a time window of 2 s and a sampling frequency of 1000 Hz. The dataset can be potentially analyzed using different techniques. However, our field setup was thought to analyze the dispersive behavior of the shallow subsurface (e.g. Multichannel Analysis of Surface Waves - MASW [5]).

**Table 2:** Main acquisition parameters of the active multichannel configurations used at the Marcite site (OpenDocument “.ods” and comma-separated values “.csv” formats are included in the supplementary material).

Label (data filename)	Geophone spacing (m)	S-G1 offset (m)
*Configuration M2*		
M2-2	2	−2
M2-4	2	−4
M2_24	2	24
M2_26	2	26
*Configuration M4*		
M4-4	4	−4
M4-6	4	−6
M4-8	4	−8
M4_48	4	48
M4_50	4	50
M4_52	4	52

### 2.2 Equipment

All the seismic vibration records (single stations and arrays) were measured using Reftek130 digitizers coupled to Le3d5s velocimeters. In some of these sites, we have co-located (i.e. at the same place) a SARA Geobox (4.5 Hz and 0.5 Hz tern of geophones) (Fig. 2). At a few other points, measurements were repeated in different time periods and slightly different positions in the order of a few meters (Table 1). All measurements using Reftek130 digitizer were provided with a GPS antenna and therefore synchronized with the UTC reference time. The measurements using SARA Geobox were recorded without a GPS antenna, and therefore are provided without UTC time synchronization. The amplitude scale of all files in the repository is a velocity in meters per second (m/s). The amplitude of time series was originally in digital counts, but for homogeneity of the dataset we prefer to store the entire data set in m/s after applying the instrumental corrections.

### 2.3 Data assembly

The passive ambient vibration measurements are provided as binary SAC format [6]. In the repository [3], the SAC binary files are named for example as 171090945_R.N01E (see Table 1), where the first part of the name indicates the time period of the recording following the scheme YYJDHHMM; where YY JD HH and MM stands for year (17 means 2017), julian day (109 in the example) and starting hours (09, UTC time) and minutes of acquisitions (45), respectively. Because we used two types of equipment, the flag _R or (_S) indicates noise recording performed with Reftek130 coupled to Le3d5s velocimeter (whilst the flag _S with a Sara Geobox). The second part of the file name after the dot refers to the code name of the temporary station (N01 in the previous example), and the last letter indicates the component of the ground motion (E, N and Z means EW, NS and UP components, respectively). Because our dataset is composed of three-components measurements, at each site we have always three files (following with the previous example 171090945_R.N01e, 171090945_R.N01n and 171090945_R.N01z). The SAC binary format is a common format in the seismological community, and it is used within the Sac software (Seismic Analysis Code, http://ds.iris.edu/files/sac-manual/; [6]), an interactive program designed for the study of seismic signals, especially time-series data. It can be requested by following the instructions on the web page accessed via the link: http://ds.iris.edu/ds/nodes/dmc/forms/sac/ (last accessed on 2020/04/04)*.* The SAC binary format is convenient with respect to ASCII format because the file size is smaller. Further the SAC binary format keeps other important information into the headers; for example, NPTS (number of samples in the time series), DELTA (sampling step in seconds; e.g. 4e−3 corresponds to 250 Hz), KZTIME (begin time in the format hour, minute, seconds and mseconds; e.g. 09:45:42.000), STLA (latitude of the measurement point in decimal degree; e.g. 4.281351e+01) and STLO (longitude; e.g. 1.311644e+01), KSTNM (station name that was set equal to the code into the name; e.g. N01) and KCMPNM (component of ground motion; e.g. E for EW component).

The SAC binary format is automatically read by other software commonly used for the analysis of seismic data, such as the opensource code geopsy (www.geopsy.org, last accessed on 04/04/2020). Geopsy is a quite standard tool to analyze passive data [7]. In any case SAC binary format can be easily converted in ascii files, using software such as the same geopsy (e.g. the command line “geopsy 171101313_R.N19E -export file_output.txt” easily converts a SAC file in a one column ascii file).

The data of the 2D arrays (MB, MS, FB and FS; acronymous for Marcite Big, Marcite Small, Fontevena Big and Fontevena Small, Fig. 1), as described in the main text of [2], keep the same format of the single-station measurements, except that the time indicated in the name does not correspond to the starting time of the stored files. This is because all the 2D array data has been already synchronized and trimmed (setting the begin header into the sac file equal to zero), and therefore the data set of each single array is ready to be processed for array analysis.

The active multichannel data are provided as SEG-Y files [8], obtained after conversion of the proprietary *drm format through the GEOEXPLORER software (Sara Electronic Instruments S.R.L.). The filename in the dataset describes basic information: for example, “M2-2” indicates, in its first part, the linear array configuration (Marcite area – M) and the geophone spacing in meters (2), whilst “−2” suggest the source (S) - geophone (G1) minimum offset in meters (an underscore divides the filename for a positive offset along the array, e.g. “M2_26”).

Together with the seismic records, we provide ancillary information represented by a Geospatial dataset provided as an open-source GIS project (EPGS: 32633) created with QGIS software (https://qgis.org/en/site/, last access April 2020). The project includes 18 vectors (EPGS: 4326) and one OpenStreetMap (OSM) basemap (EPGS: 3857). In addition, we provide each layer as separate Geopackage (*.gpkg) and Google **K**eyhole **M**arkup **L**anguage (*.kml) files. This geospatial dataset contains the location and geometry of the seismic surveys carried out at the Norcia basin, together with some layers related to the paper [2].

The layer NOI (cyan points) includes all the points of single-station measurements recorded with the Reftek and Sara equipment. The layers FB, FS, MB and MS report the location of the four helical arrays [2]. The orange points report the two big arrays (Fontevena Big - FB) and (Marcite Big - MB); the green points display the two small arrays, respectively (Fontevena Small - FS and Marcite Small - MS). The three vectors “Section_S1, Section_S2, Section_S3” and the layer “Velocity_mod_boundary” are the cross-sections and the velocity models boundary by [2]. The layer groups M2 and M4 gather the information related to the active surveys. The vectors M2_G1-G12 and M4_G1G12 represent shorter and longer linear seismic arrays, respectively (the geophone G1 to the North and G12 Southward). The starting and end points vectors of each one are displayed by vectors with “filename_p” (e.g. M2_G1-G12). The position of the seismic sources is also reported as red point vectors (e.g. filename “S_M2_G1”), labelled with the minimum offset information. A Web Map Service (WMS) layer from OpenStreetMap is also provided as a basemap [9;10]. The service is freely available from the website (www.openstreetmap.org, last access April 2020) and is integrated in the QGIS project through the OpenLayers plugin (//plugins.qgis.org/plugins/openlayers_plugin/, last access April 2020).

## Declaration of Competing Interest

The authors declare that they have no known competing financial interests or personal relationships which have, or could be perceived to have, influenced the work reported in this article.

## Appendix Supplementary materials

Image, application 1

Image, application 2
